# Reducing acrylamide in bread with plant additives: a systematic review^☆^

**DOI:** 10.1016/j.fochx.2025.103378

**Published:** 2025-12-05

**Authors:** Amirhossein Abedini, Mahla Salimi, Azizollah Zargaraan, Parisa Sadighara, Mahmood Alizadeh Sani, Amir M. Mortazavian, Abdorreza Mohammadi

**Affiliations:** a Student Research Committee, Department of Food Science and Technology, Faculty of Nutrition Science and Food Technology, National Nutrition and Food Technology Research Institute, Shahid Beheshti University of Medical Sciences, Tehran, Iran; b Institute of Human Nutrition and Food Science, Kiel University, Heinrich-Hecht-Platz 10, 24118 Kiel, Germany; c Department of Food and Nutrition Policy and Planning Research, National Nutrition and Food Technology Research Institute and Faculty of Nutrition and Food Science, Shahid Beheshti University of Medical Sciences and Health Services, Tehran, Iran; d Division of Food Safety and Hygiene, Department of Environmental Health Engineering, School of Public Health, Tehran University of Medical Sciences, Tehran, Iran; e Department of Food Science and Technology, School of Nutritional Sciences and Dietetics, Tehran University of Medical Sciences, Tehran, Iran; f Nutraceutics Research Center, Tehran University of Medical Sciences, Tehran, Iran; g Department of Food Science and Technology, National Nutrition and Food Technology Research Institute, Faculty of Nutrition Sciences and Food Technology, Shahid Beheshti University of Medical Sciences, Tehran, Iran

**Keywords:** Plant extracts, Plant powders, Acrylamide, Maillard reaction

## Abstract

Reducing acrylamide (AA) formation in thermally processed foods, particularly bread, is a major food safety concern. Plant-based additives, including powders and extracts, offer a promising natural strategy for AA mitigation due to their bioactive compounds, such as polyphenols and flavonoids, which can scavenge reactive intermediates, modulate Maillard reactions, and inhibit lipid oxidation. This study systematically evaluated the effects of various plant powders and extracts, including green tea, rosemary, turmeric, buckwheat, and traditional spices, on AA formation in bread. Based on reported studies, incorporation of plant extracts such as green tea, vine tea, sorghum bran, rosemary, and Tartary buckwheat into baked and fried products resulted in acrylamide reduction ranging from 2.7% (0.5 g/kg green tea) to 97.1% (1500 mg/kg Caesalpinia spinosa), depending on the type, concentration, and food matrix used. Conversely, certain powders, such as chia seeds, garlic, Thymus vulgaris, and Carum carvi, have been found to enhance AA formation because of their carbohydrate content or specific reactive compounds. Processing parameters, including baking temperature, duration, and fermentation, also strongly influence AA outcomes. Overall, plant extracts generally outperformed powders in terms of AA mitigation, highlighting the importance of additive selection and optimization. These findings provide critical insights into the development of safer bakery products and suggest that combined strategies, including the use of antioxidants and fermentation, can maximize acrylamide reduction.

## Highlights

• Plant extracts like green tea, rosemary, and turmeric reduce acrylamide formation in bread via bioactive compounds.
• Certain natural additives, such as chia seeds and garlic, may increase acrylamide levels in bread.
• Bioactive compounds modify the Maillard reaction, influencing acrylamide precursor interactions.
• Future meta-analyses are recommended to further elucidate the contradictory effects of plant-based additives.

## 1 Introduction

**Fig. 1:**
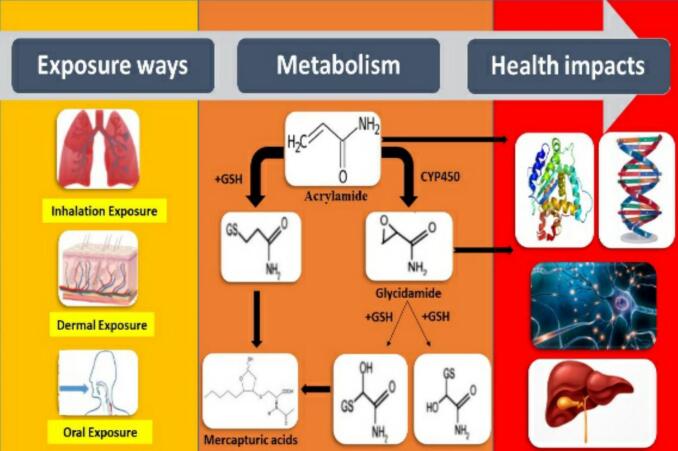
Overview of Acrylamide exposure and its health impacts.

High-temperature cooking processes such as frying, baking, and roasting produce chemical compounds like acrylamide (AA), which has potential health risks (Abedini et al., 2024). The International Agency for Research on Cancer has identified AA as a possible carcinogen in a report (Adimas, Abera, Adimas, Woldemariam, & Delele, 2024; Pandiselvam et al., 2024). Experimental studies on animals and evidence from humans suggest that AA is neurotoxic, although its exact mechanism of action remains unclear (Lindeman et al., 2021). Some research points to oxidative stress in brain tissues as a key factor in its neurotoxicity, leading to reduced levels of glutathione and decreased activity of Glutathione S-transferases (Farouk, Gad, Almeer, Abdel-Daim, & Emam, 2021). Additionally, AA has been shown to cause death of cerebellar Purkinje cells. Other studies suggest that it lowers levels of neurotransmitters like dopamine, serotonin, and norepinephrine, contributing to depression in some cases (Dasari, Ganjayi, & Meriga, 2018; Faria et al., 2018). AA also induces mitochondrial collapse and triggers apoptosis in both the peripheral and central nervous systems, resulting in neuronal destruction (Rahbardar, Farmad, Hosseinzadeh, & Mehri, 2021). There is also evidence that it reduces brain creatine phosphokinase activity, leading to Adenosine Triphosphate (ATP) depletion and further apoptosis, a widely accepted mechanism in AA's neurotoxicity (Kopanska, Muchacka, Czech, Batoryna, & Formicki, 2018) (figure 1). AA formation in foods and its subsequent exposure in humans represent a critical link between food-processing chemistry and public health. Figure 1 provides an overview of AA exposure pathways, metabolic transformation, and associated toxicological impacts. Upon ingestion, acrylamide is rapidly absorbed and metabolized to glycidamide, a reactive epoxide that can bind to DNA and proteins, thereby contributing to genotoxicity, neurotoxicity, and potential carcinogenicity. The figure also summarizes key organ targets (e.g., nervous system, reproductive organs, and liver) and highlights epidemiological findings linking dietary acrylamide exposure with increased risks of cancer and neurological disorders (Faria et al., 2018).

Therefore, legislative food bodies emphasize the need for education and awareness for people to choose the right food and reduce the formation of AA in food products in the food industry. The removal of AA through the Maillard reaction in food is challenging, one of which has a serious impact on sensory properties. For many years, researchers have focused on the use of food additives to reduce AA in high-consumption products such as bread (Akbari-Adergani et al., 2022; Govindaraju et al., 2024). Statista reports that by 2029, bread turnover will reach $0.56 trillion, up from $0.36 trillion in 2018. In addition, China, India, America, Japan, and Germany are the five countries that consume the most bread by 2024 (Statista, 2024). The use of plant-based products, such as extracts and powders (rich in bioactive compounds such as polyphenols, flavonoids, and other antioxidants), can be effective in reducing the formation of AA and improving the nutritional properties of foods (Abedini et al., 2024). Due to their high antioxidant capacity, bioactive compounds can be effective in the complex and diverse spectrum of AA formation reactions, such as direct reaction with AA precursors, browning of foods and AA formation, and modification of the reaction conditions (Abedini et al., 2022). The interaction between reducing sugars and amino acids, especially asparagine, leads to the Maillard reaction, which is the first step in AA formation. Plant extracts can interfere effectively with these compounds and reactions due to bioactive extracts and effective functional groups (Govindaraju et al., 2024; Liu et al., 2015; Morales, Jimenez, Garcia, Mendoza, & Beristain, 2014). Some studies have shown that free radicals are neutralized by polyphenols in plant extracts, or the presence of some enzymes in plant matrices, such as L-asparaginase, can neutralize the first step of Maillard formation (Ciesarová & Kukurová, 2024). Another less-evaluated effect is the effect of plant extracts on Maillard completion conditions, such as pH and water activity. The influencing factors in this matter are the composition of the food matrix and the concentration of plant materials used. Investigations have shown that the profile of natural substances in each plant extract can have a different effect on Maillard formation reactions owing to the difference in antioxidant capacity (Oral, Dogan, & Sarioglu, 2014). The premise of researchers is the formation of AA, but sometimes using more than the limit by choosing inappropriate natural resources can lead to an increase in its formation. Some studies have shown that the use of chia seeds, garlic, *Thymus vulgaris*, and *Carum carvi* in bread can significantly increase AA levels. Therefore, it is very important to understand the use of optimal formulation of plant extracts required for effective reduction of AA in bread (Abedini, Sadighara, Sani, & McClements, 2023; Fan et al., 2023). Because of conflicting studies, this study aimed to investigate the effect of plant extracts and powders on the formation of AA in bread. This study discusses aspects of the increasing or decreasing effects to report a correct understanding of the effect of natural sources on the formation of AA in bread. Examining the evaluation techniques of AA, the mechanisms of the effect of bioactive compounds, and the future of research in this field are the other goals of this study.

## 2 Mechanism of active Compounds on Acrylamide Formation

At temperatures of 120 °C and above, the reaction between reducing sugars and the amino acid asparagine leads to the formation of AA through the Maillard reaction (Verma & Yadav, 2024). One effective approach to prevent AA formation in bread involves the use of active compounds such as plant extracts and powders, which can influence AA production through various mechanisms. One such mechanism is the direct interaction with Maillard reaction precursors, including asparagine or reducing sugars (Hamad et al., 2024). Plant-derived materials contain polyphenols and enzymes that are capable of forming non-reactive complexes with asparagine, thereby modulating the Maillard reaction. Another mechanism involves the impact of reactive intermediates, such as free radicals and carbonyl compounds (Hamzalıoğlu & Gökmen, 2024). The neutralization of these intermediates depends largely on the antioxidant capacity of the plant substances. Plants are generally rich in bioactive compounds such as polyphenols, flavonoids, and vitamins that can effectively scavenge free radicals and reactive carbonyls (Hamzalıoğlu & Gökmen, 2024). A further mechanism is associated with enzymatic inhibition. Certain plant sources, such as *Pisum sativum,* contain L-asparaginase, an enzyme that converts asparagine into aspartate, thereby markedly reducing the progression of the Maillard reaction (Aiswarya, Baskar, Naveenkumar, & Pravin, 2024). Another possible mechanism involves the formation of nontoxic complexes between AA and plant-derived compounds. These complexes can adsorb or bind AA within food matrices, leading to a decrease in their concentration (Peivasteh-Roudsari et al., 2024).

## 3 Additives that increase AA formation

Food additives do not always reduce the formation of AA; therefore, researchers and industry professionals must select them with caution. Products containing additives such as cocoa and corn syrup are particularly susceptible to elevated AA levels, especially under certain temperature conditions and extended roasting durations (Farah, Zaibunnisa, Misnawi, & Zainal, 2012; Sarion, Codină, & Dabija, 2021). For instance, one study reported that cocoa significantly contributes to AA formation due to the high heat applied during cocoa bean roasting and other thermal processing steps (Ofosu, Ankar-Brewoo, Lutterodt, Benefo, & Menyah, 2019). Similarly, the addition of cocoa to biscuits was found to increase AA concentrations, primarily because of the roasting involved in the manufacturing process (Suman, Generotti, Cirlini, & Dall’Asta, C., 2019). Recent investigations have also examined the influence of varying fructose concentrations in high-fructose corn syrup incorporated into biscuits, revealing a substantial rise in AA content with increasing fructose levels (Ershadi, Azizi, & Najafian, 2021). Notably, certain synthetic additives, including acrylic acid, N-acetyl-glucosamine, and glucosamine, tend to promote AA formation, as reported in several studies. Charoenprasert, Zweigenbaum, Zhang, and Mitchell (2017) observed that these compounds significantly increased AA levels in black ripe olives. The presence of multiple reactive functional groups such as carbonyl, hydroxyl, and amine groups likely contributed to this effect. Among these, glucosamine demonstrated the most pronounced effect, causing a 129% increase in AA formation (Charoenprasert et al., 2017). Conversely, some substances have been shown to exert no significant effect on AA levels. For example, the addition of calcium chloride (CaCl₂) and sodium chloride (NaCl) to green ripe olives, incorporation of garlic into cereal crisps, and use of caramel or molasses (5 g/kg) in non-centrifugal cane sugar (NCS) did not significantly alter AA concentrations (Jaworska et al., 2019; Martín-Vertedor et al., 2020; Sung, Chi, Chiou, Lin, & Lee, 2020). Moreover, differences in the physical forms of the additives can influence their effects on AA formation. For instance, some herbal additives in the extract form significantly reduce AA formation, whereas their powdered counterparts have been reported to increase it. Despite these findings, the potential of food additives to enhance AA formation has not been widely addressed. Considering the widespread use of food additives in high-consumption products, such as bread, future research should focus on determining optimal formulations and dosages to effectively manage AA formation in food products.

## 4 Mitigation strategies of AA in bread

AA levels in bread vary according to several factors, including processing time and temperature, the concentration of AA precursors in grains, grain variety and composition, fermentation duration, and the co-ingredients incorporated into the formulation. Free asparagine, when combined with reducing sugars at elevated temperatures (>120 °C), serves as a major contributor to AA formation in cereal grains (Mottram, Wedzicha, & Dodson, 2002; Stadler et al., 2002). The concentration of free asparagine differs among cereal species. Žilić et al. (2017) reported that average free asparagine concentrations in cereals ranged from 426 ± 144 to 1179 ± 359 mg/kg, with rye and oats exhibiting higher levels than wheat, thereby predisposing them to greater AA formation (Fredriksson, Tallving, Rosen, & Åman, 2004; Žilić et al., 2017). Previous studies have shown that rye bread typically contains higher AA levels than wheat bread due to its elevated asparagine content. Although free asparagine is not the sole precursor of AA, it plays a pivotal role in determining AA concentrations in cereal-based baked products (Boyacı Gündüz & Cengiz, 2015). A study by Çelik and Gökmen (2020) revealed that whole rye fermented dough contained high levels of free asparagine (7585 ± 158 mg/kg), resulting in the highest AA concentrations (1565–3887 μg/kg) in the crust of rye bread compared to other cereals. The specific grain components used in bread formulation also affect AA formation, as free asparagine is concentrated primarily in the germ and bran. Consequently, utilizing grains with lower asparagine content, or reducing the proportion of bran and germ, could lower AA levels in bread. However, such adjustments may alter the bread’s flavor, texture, and nutritional quality key attributes influencing consumer preference. Therefore, reducing AA formation without compromising sensory and nutritional properties remains a significant challenge, particularly for high-bran breads valued for their fiber and health benefits (Boyaci Gunduz, 2023). Lowering the flour extraction rate can also decrease the asparagine concentration in flour, although this approach may lead to economic drawbacks due to reduced flour yield (Malunga et al., 2019). Additional formulation-stage strategies to minimize AA formation include the application of asparaginase or the evaluation of alternative co-ingredients (Boyaci Gunduz, 2023). Nevertheless, incorporating other flours, grains, nuts, or dried fruits can inadvertently elevate AA levels, as these components may contain higher amounts of AA precursors. Fortified breads containing nuts and seeds often exhibit increased AA concentrations, particularly when these ingredients are roasted prior to incorporation. To mitigate this, nuts and seeds may be roasted at lower temperatures, or ingredients rich in fructose could be substituted with those containing more glucose (Boyaci Gunduz, 2023). Optimization of processing parameters such as baking temperature, temperature profile, and duration is critical to minimizing thermal exposure and thereby reducing AA levels in bread (Onacik-Gür, Szafrańska, Roszko, & Stępniewska, 2022). A recent study demonstrated that reducing the baking temperature from 260 °C to 230 °C decreased AA content in rye bread by approximately 25% (Onacik-Gür et al., 2022). Baking to achieve a lighter crust color is another effective approach, as AA is primarily localized in the crust, with only trace amounts present in the crumb due to lower heat exposure and higher moisture content. A strong correlation has been established between crust coloration and AA concentration, highlighting the importance of avoiding dark or burnt crusts (Mesias, Delgado-Andrade, & Morales, 2022). Extending dough fermentation time is another practical method to reduce AA formation, as yeast metabolizes precursors such as asparagine (Mesias et al., 2022). Fermentation involving both yeast and lactic acid bacteria can further decrease AA content by reducing dough pH, particularly in sourdough breads (Nachi et al., 2018). Sourdough fermentation enhances the dough’s functional properties through microbial metabolism, and breads fermented with selected lactic acid bacteria strains alongside yeast exhibit significantly lower AA levels than those fermented with yeast alone (De Vuyst, Comasio, & Kerrebroeck, 2023). This reduction is primarily attributed to glucose metabolism and pH decrease resulting from lactic acid production. A study on wheat breads fermented with combinations of *Pediococcus pentosaceus, Limosilactobacillus fermentum*, and *Saccharomyces cerevisiae* demonstrated a 24.38% to 58.83% reduction in AA levels due to increased acidity and diminished precursor availability (Zhou et al., 2022). The extent of AA reduction varies according to microbial strain and environmental conditions such as reactant availability and pH. Lactic acid bacteria exhibiting low amylolytic, but high proteolytic activity are particularly effective in minimizing AA formation in bread (Bartkiene et al., 2013). Acrylamide formation in thermally processed foods is a recognized public health concern due to its probable carcinogenicity. Regulatory authorities, including EFSA and WHO/FAO, have recommended multiple mitigation strategies to reduce dietary exposure. These strategies include the selection of raw materials with low levels of reducing sugars and asparagine, pre-processing treatments such as soaking or blanching, and optimization of processing conditions including temperature, time, and moisture. The application of enzymes, particularly asparaginase, has been shown to effectively reduce acrylamide formation in bakery and cereal products. In addition, color-based endpoint monitoring during cooking and proper storage of raw materials are practical measures endorsed by both regulatory and industry guidelines. Collectively, these approaches aim to minimize acrylamide exposure in accordance with the ALARA (as low as reasonably achievable) principle (Abedini et al., 2023).

## 5 Methods

According to the entry and exit criteria and the Prisma checklist, this systematic review article was conducted by two researchers.

### 5.1 Search strategy

According to the keywords (AA or Acrylamide) and (mitigation or reduction or decrease) and (extract or "plant extract" or "plant powder") and (bread) databases of PubMed, Science Direct, Web of Science, and Scopus were used to search for English articles. The search date was June 26 and there was no time limit. 395 titles and abstracts were obtained from all databases and were reviewed by three researchers based on input and output criteria.

### 5.2 Inclusion and exclusion criteria

A formal protocol for this review was not registered. However, to ensure transparency and reproducibility, detailed inclusion and exclusion criteria were established prior to the literature search. Studies were included if they investigated the effects of plant-based additives, extracts, or powders on acrylamide formation in bread or bakery products, were original research articles including experimental studies or controlled trials, were published in English, reported quantitative or qualitative measurements of acrylamide content, and used clearly defined methodologies for acrylamide detection such as GC-MS, LC-MS/MS, HPLC, or ELISA. Studies were excluded if they were not related to bread or bakery products, were reviews, meta-analyses, conference abstracts, editorials, or commentaries without original data, had insufficient methodological details for acrylamide measurement, were not published in English, or were duplicate publications or overlapping datasets.

### 5.3 Data extraction

Relevant information was extracted by two researchers in Table 1. If there was any ambiguity or question in the review of the articles, the third author was consulted.

**Table 1:** Information about impacts of plant extracts and powders on AA formation in bread.

Country	Incorporation type	Plant type	Concentration	Food product	Detection method	Increase or decrease of acrylamide (%)	References
China	Extract	Vine Tea (Ampelopsis grossedentata)	1.25 g/kg	Wheat bread	UHPLC-ESI-HRMS/MS	-58.23	(Ma et al., 2020)
			2.5 g/kg			-33.32	
	Extract	Dihydromyricetin	9.97 mg/kg	Wheat bread	UHPLC-ESI-HRMS/MS	-32.43	
			19.94 mg/kg			-18.31	
China	Extract	Green tea	0.002 g/kg	Fried bread sticks	LC-MS/MS	-24	(Zhang & Zhang, 2007)
			0.01 g/kg			-28.8	
			0.1 g/kg			-72.5	
			1 g/kg			-46.6	
			2.5 g/kg			-28.8	
			4.9 g/kg			-6.6	
	Powder	Bamboo leaves	0.002 g/kg	Fried bread sticks	LC-MS/MS	-10	
			0.01 g/kg			-40	
			0.1 g/kg			-67.5	
			1 g/kg			-82.9	
			2.5 g/kg			-65.0	
			4.9 g/kg			-30	
New Zealand	Extract	(-)-epigallocatechin gallate (EGCG) extracted from green	3.3 g/kg	White bread	LC-MS	-31.1	(Fu et al., 2018)
			6.6 g/kg			-35.3	
			9.9 g/kg			-38.4	
China	Extract	Tartary buckwheat seed	253 μg L−1	Wheat bread	GC	-23.5	(Jing et al., 2019)
		Tartary buckwheat sprouts	195 μg L−1			-27.3	
		Common buckwheat seed	261 μg L−1			-17.0	
		Common buckwheat sprouts	265 μg L−1			-16.7	
Saudi Arabia	Extract	Spearmint (Mentha spicata), Fennel (Foeniculum vulgare) and Turmeric (Curcuma longa)	25 g/kg	Pita breads	HPLC	-89	(Namir, Rabie, Rabie, & Ramadan, 2018)
			14 g/kg			-59.5	
			3 g/kg			-71.8	
Iraq	Extract	Green tea	3 g/kg	Bread crust	HPLC	-57.7	(Mousa, Khalid, & Hamood, 2019)
		Thyme	3 g/kg			-70.1	
		Garlic	3 g/kg			-59.8	
		Anise	3 g/kg			-65	
	Powder	Cinnamon	3 g/kg			+103.5	
USA	Extract	Sorghum bran	0.5 g/kg	Wheat bread	UPLC-DAD-ESI-Q-TOF-MS/MS	-53.04	(Chen, Wang, & Li, 2022)
			1.0 g/kg			-60.8	
			1.5 g/kg			-69.9	
		Grapeseed	0.5 g/kg	Wheat bread	UPLC-DAD-ESI-Q-TOF-MS/MS	-40.1	
			1.0 g/kg			-52.03	
			1.5 g/kg			-77.7	
		Green tea	0.5 g/kg	Wheat bread	UPLC-DAD-ESI-Q-TOF-MS/MS	-2.7	
			1.0 g/kg			-10.8	
			1.5 g/kg			-68.9	
Poland	Extract	Green tea	0.1 g/kg	Rye bread	GC/MS	+42.4	(Onacik-Gür et al., 2022)
			0.5 g/kg			+57.5	
New Zealand	Extract	Green tea	3.3 g/kg	White bread	LC-MS	-30.24	(Fu, 2016)
			6.6 g/kg			-34.30	
			9.9 g/kg			-37.36	
Romania	Extract	Rosemary	0.02		HPLC-UV	Compared to the control sample the AA levels decreases for the samples with antioxidant addition	(Sarion, Dabija, & Codină, 2021)
			0.1				
			0.5				
Portugal	Extract	Rocha Pear Peel	Dehydrated extract	Wheat bread	UPLC-MS/MS	The biggest decrease in traditional oven with 19.2% is related to dehydrated extract	(Morgado et al., 2019)
			Aqueous extract				
			Dehydrated extract	Rye bread	UPLC-MS/MS		
			Aqueous extract				
Chile	Extract	Tara pod (Caesalpinia spinosa)	500 mg/kg	Chilean bread		-18.07	(Pedreschi et al., 2018)
			600			-31.3	
			750			-45.7	
			1000			-63.8	
			1500			-97.12	
Egypt	Powder	Thyme	3 g/kg	Arabic bread	HPLC	-98.2	(Tesby, Neveen, Neveen, & Nashwa, 2018)
		Cumin	3 g/kg			-92.8	
		Anise	3 g/kg			-100	
		Thyme	3 g/kg	Patonsalé	HPLC	-24.3	
		Cumin	3 g/kg			-7.3	
		Anise	3 g/kg			-26.8	
		Thyme	3 g/kg	Cressina	HPLC	-22.8	
		Cumin	3 g/kg			-38.5	
		Anise	3 g/kg			-45.7	
Italy	Powder	Chia seeds (Salvia hispanica L.)	2 g/kg	Wheat bread	LC-MS/MS	+22.3	(Galluzzo et al., 2021)
			5 g/kg			+27.7	
			7 g/kg			+8.2	
			10 g/kg			+22.9	
Poland	Powder	Garlic	0.75 g/kg	Crispbread	HPLC	+5.1	(Jaworska et al., 2019)
			1.5 g/kg			+9.3	
		Ginger	0.75 g/kg			+6.26	
			1.5 g/kg			+23	
Russia	Powder	Blueberry	3 g/kg	Wheat bread 4% fat -220 °С - Crust	Electrophorese	-10.6	(Nilova, Malyutenkova, & Kruchina-Bogdanov, 2019)
		Pine nut	6 g/kg			-30.3	
		Sea buckthorn	5 g/kg	Wheat bread 14% fat-220 °С		-24.3	
		Rowan	5 g/kg			-17.4	
		Blueberry	3 g/kg	Wheat bread 4% fat -200 °С		-22.7	
		Pine nut	6 g/kg			-33.5	
		Sea buckthorn	5 g/kg	Wheat bread 14% fat-200 °С		-34.1	
		Rowan	5 g/kg			-24.7	
Lithuania	Powder	Thymus vulgaris	5 g/kg	Wheat bread ( with scalded flour)	LC-MS/MS	+74.12	(Bartkiene et al., 2018)
			10 g/kg			+241.1	
			15 g/kg			+288.2	
		Carum carvi	5 g/kg			+131.7	
			10 g/kg			+184.7	
			15 g/kg			+277	
		Origanum vulgare	5 g/kg			+76.4	
			10 g/kg			+71.7	
			15 g/kg			+155.2	
		Ocimum basilicum	5 g/kg			+32.9	
			10 g/kg			+102.3	
			15 g/kg			+189.4	
		Coriandrum sativum	5 g/kg			+67	
			10 g/kg			+124.7	
			15 g/kg			+201.1	
Lithuania	Powder	Thymus vulgaris	5 g/kg	Wheat bread (fermented with *L. plantarum*)	LC-MS/MS	+9.4	
			10 g/kg			+163.5	
			15 g/kg			+9.4	
		Carum carvi	5 g/kg			+68.2	
			10 g/kg			+50.5	
			15 g/kg			+81.1	
		Origanum vulgare	5 g/kg			-29.4	
			10 g/kg			+4.7	
			15 g/kg			+43.1	
		Ocimum basilicum	5 g/kg			-4.7	
			10 g/kg			-4.7	
			15 g/kg			+72.9	
		Coriandrum sativum	5 g/kg			+38.8	
			10 g/kg			+28.2	
			15 g/kg			-40	

### 5.4 Results

**Fig. 2:**
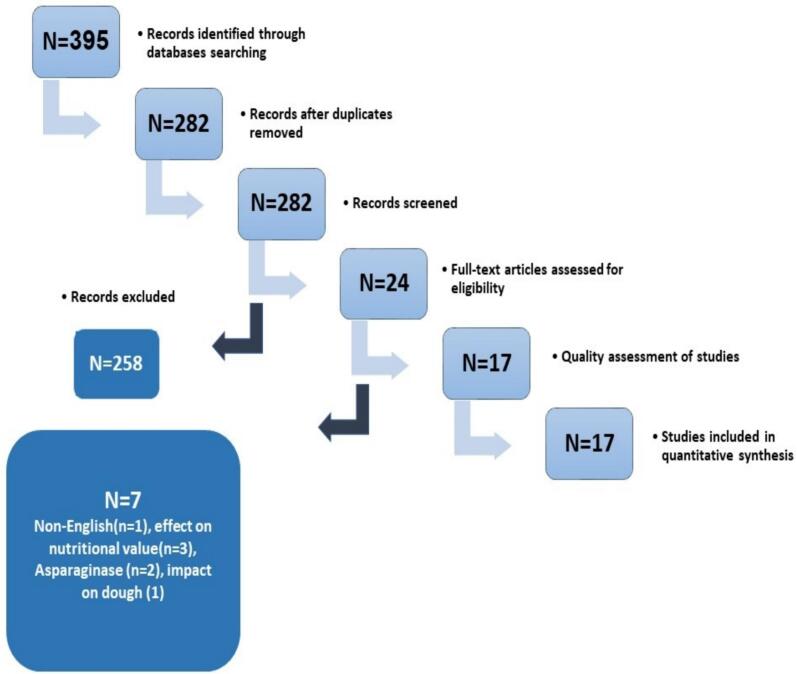
Diagram of study.

From the search in the mentioned databases, 395 articles were obtained. After removing the duplicates, 282 articles were reviewed. Articles that did not meet the inclusion criteria were excluded from the study, and 24 articles remained for full-text review. Finally, according to the entry criteria, 17 articles were suitable for inclusion in the article. Figure 2 shows the Prisma checklist.

### 5.5 The descriptive results of screened manuscripts

17 articles were selected to be included in the systematic article and the extracted data included country, type of addition, type of plant, concentration of additive, type of bread, detection method, increasing or decreasing effect and references.

## 6 Discussion

AA analysis in food products is typically performed using advanced analytical techniques such as gas chromatography–mass spectrometry (GC–MS), liquid chromatography–tandem mass spectrometry (LC–MS/MS), High-performance liquid chromatography (HPLC), and enzyme-linked immunosorbent assay (ELISA). In this section, the effects of plant extracts and powders on AA formation in bread are discussed, with emphasis on their mechanisms of action and efficiency in reducing AA concentrations.

### 6.1 Extracts

**Fig. 3:**
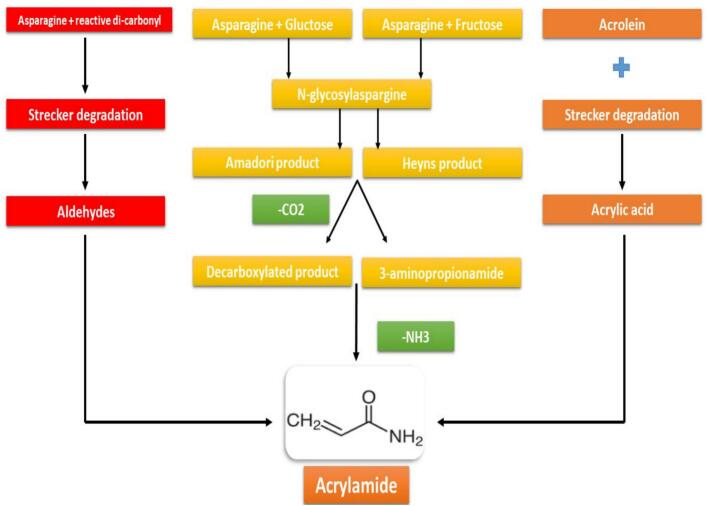
Acrylamide formation in food products.

The impact of plant extracts on acrylamide formation in bread has gained significant attention as a natural and sustainable approach to mitigate this harmful compound. Acrylamide is primarily produced during baking when asparagine, an amino acid, reacts with reducing sugars at elevated temperatures through the Maillard reaction an essential process in bread production that also contributes to flavor and color development. Plant extracts derived from sources such as rosemary, turmeric, and green tea have demonstrated the ability to reduce acrylamide formation due to their rich content of bioactive compounds. For instance, rosemary contains rosmarinic acid and carnosic acid, which exhibit strong antioxidant properties capable of inhibiting the Maillard reaction and thereby limiting AA production (Sarion, Dabija, & Codină, 2021). Similarly, green tea is abundant in catechins, particularly epigallocatechin gallate (EGCG), which can reduce AA formation by scavenging free radicals and interfering with the Maillard reaction pathway (Fu, 2016). Ma et al. (2020) investigated the effect of hot-water vine tea extract as an exogenous additive on AA formation and bread quality. The addition of 1.25 g/kg vine tea extract resulted in a 58.23% decrease in AA content. This reduction was attributed to the antioxidant activity of polyphenols, particularly *dihydromyricetin*, present in the extract. However, increasing the dosage of the extract yielded diminishing effects, likely due to the formation of hydrogen peroxide, which may promote AA generation during baking. The researchers further examined *dihydromyricetin* individually and observed a 32.43% reduction in AA at an optimal concentration of 9.97 mg/kg. The antioxidant activity of polyphenols, including their free-radical scavenging capacity and inhibition of lipid oxidation, plays a key role in mitigating AA formation. Importantly, sensory evaluation results indicated that the addition of vine tea extract did not adversely affect bread acceptability (Ma et al., 2020). In another study, the effects of bamboo leaf extract (AOB) and green tea extract (EGT) on AA reduction in fried breadsticks were evaluated. The incorporation of AOB and EGT significantly decreased AA levels, achieving reductions of 72.5% and 82.9% at concentrations of 0.1 and 1 g/kg, respectively. Sensory analyses revealed no significant differences in texture or flavor compared to the control samples. These findings suggest that yeast fermentation prior to deep-frying contributes to lower AA formation by reducing the availability of free asparagine and sugars, which are key precursors in the Maillard reaction. Similar to the aforementioned findings, a strong correlation was observed between the anti-oxidative activity of these extracts and the extent of AA reduction. Furthermore, an additional mechanism was proposed involving the acrolein pathway, which forms acrylamide through lipid oxidation and degradation of glycerol and fatty acids. The addition of antioxidants was shown to inhibit AA formation via this pathway by scavenging free radicals and ions and by preventing lipid oxidation (Zhang & Zhang, 2007) (Figure 3). Figure 3 illustrates the principal chemical pathways involved in AA formation during thermal processing of carbohydrate-rich foods. The predominant mechanism originates from the Maillard reaction between free asparagine and reducing sugars, such as glucose or fructose, leading to the formation of N-glycosylasparagine intermediates. These intermediates undergo subsequent Amadori and Heyns rearrangements, producing decarboxylated and deaminated products (e.g., 3-aminopropionamide), which ultimately degrade into AA. Additionally, the acrolein pathway, arising from lipid oxidation and Strecker degradation, contributes to AA formation through the conversion of acrolein into acrylic acid and subsequent condensation with ammonia. The figure highlights how both sugar–amino acid reactions and lipid-derived aldehydes converge to yield acrylamide, emphasizing the multifactorial nature of its formation in thermally processed foods. Understanding these pathways is essential for designing targeted mitigation strategies that inhibit key precursors or reaction intermediates (Abedini et al., 2024).

EGCG, one of the major bioactive components of green tea extract (GTE), has been extensively investigated for its role in reducing AA formation during baking. Fu et al. (2018) examined the influence of EGCG on AA generation in white bread and found that EGCG, acting as an antioxidant and free radical scavenger, effectively suppressed AA formation under constant baking conditions. Although a linear relationship between EGCG concentration and AA reduction could not be established, increasing EGCG levels consistently led to lower AA contents in the final product. Texture analysis indicated that most quality parameters remained unchanged, except for cohesiveness, as EGCG-pretreated bread samples were slightly firmer than the control group (Fu et al., 2018).

In another study, GTE was applied in combination with sorghum bran extract (SBE) and grape seed extract (GSE) at concentrations of 0.5, 1.0, and 1.5 g/kg to evaluate their combined impact on AA formation in bread. The fortified breads, rich in reactive oxygen scavengers, demonstrated significant mitigation of oxidative stress. Among the tested extracts, SBE achieved the greatest reduction in AA levels (up to 70%), correlating with its high flavonol content based on polyphenolic profile analysis (Chen et al., 2022).

The influence of GTE on rye bread was also investigated under different dough preparation conditions, including direct addition of lactic acid and the use of a freeze-dried starter culture for indirect fermentation. The results demonstrated that dough pH strongly affected by the presence of acids or yeasts had a more pronounced effect on AA mitigation than the addition of green tea extract itself (Onacik-Gür et al., 2022). Similarly, Fu (2016) reported that GTE addition effectively reduced AA formation in bread without adversely affecting texture, except for a slight increase in hardness, consistent with previous studies. Furthermore, GTE addition produced a marginally brighter crumb color. Increasing concentrations of GTE (3.3, 6.6, and 9.9 g/kg) decreased AA content by 30.24%, 34.30%, and 37.36%, respectively (Fu, 2016).

Buckwheat represents another promising antioxidant source for reducing AA in bread. Buckwheat seed and sprout extracts contain high levels of polyphenolic compounds with strong antioxidant potential (Giménez-Bastida, Zielinski, Piskula, Zielinska, & Szawara-Nowak, 2017). In a comparative study, Tartary buckwheat seeds and sprouts, as well as common buckwheat counterparts, were incorporated into bread formulations. The greatest reduction in AA (27.3%) was achieved with Tartary buckwheat sprout extract, followed by Tartary buckwheat seed extract, which reduced AA by 23.5%. The authors attributed these effects to the polyphenols’ ability to inhibit interactions between reducing sugars and asparagine the primary AA precursors by reacting preferentially with reducing sugars. The resulting bread exhibited a slightly lighter color, consistent with previous reports, and no significant differences in texture or sensory acceptance were observed (Jing et al., 2019).

Mousa et al. (2019) also investigated the influence of various plant extracts, including anise, garlic, thyme, cinnamon, and green tea, on AA formation in crusted bread. Each extract was incorporated at a concentration of 3%. The results revealed that all treated breads exhibited improved sensory characteristics, flour quality, and loaf volume, with reductions in AA levels for all treatments except cinnamon (Mousa et al., 2019).

The observation that cinnamon and nutmeg can increase AA production during baking presents an intriguing paradox, considering the well-documented antioxidant properties of cinnamon. Cinnamon is rich in phenolic compounds and flavonoids, which are generally recognized for their ability to reduce acrylamide formation by mitigating oxidative stress and inhibiting the Maillard reaction. However, despite these bioactive constituents, cinnamon’s potential to decrease AA formation appears to be counteracted by its high content of carbohydrates and aldehyde compounds, particularly *cinnamaldehyde*.

*Cinnamaldehyde*, the principal component of cinnamon essential oil, constitutes approximately 75% of the oil and is an aldehyde known to participate in chemical reactions that can promote AA formation. Furthermore, the high carbohydrate content in cinnamon (79% in the essential oil) supplies additional reducing sugars, which, when combined with asparagine under high-temperature baking conditions, can intensify the Maillard reaction and consequently increase acrylamide levels (Aghvami et al., 2023; Rannou, Laroque, Renault, Prost, & Sérot, 2016). This underscores the complexity of plant extract interactions during food processing, wherein both beneficial and pro-reactive components influence the final acrylamide outcome. Therefore, although cinnamon exhibits antioxidant potential, its elevated *cinnamaldehyde* and carbohydrate contents may counteract these advantages by fostering reaction pathways conducive to AA formation. For instance, Aghvami et al. (2023) reported that the highest acrylamide concentration was observed in cake samples containing 212.28 ng/g of cinnamon (Aghvami et al., 2023).

Morgado et al. (2019) explored another natural-based additive by incorporating plant extracts into white bread (WB) and rye bread (RB) the two most commonly consumed bread types in Portugal and baking them using both traditional ovens (TO) and conventional ovens (CO) to assess their impact on AA mitigation. Their results revealed a 27.3% reduction in acrylamide content in bread baked in a CO and a 19.2% reduction in TO (Morgado et al., 2019).

Similarly, Tara (*Caesalpinia spinosa*) pod extract was tested for its ability to mitigate acrylamide formation during the baking of “*Hallellulla* or *Hallulla*,” a traditional Chilean bread. Due to its strong antioxidant capacity and moderate reducing sugar content, tara extract effectively inhibited acrylamide formation by interfering with Maillard reaction intermediates. The addition of 750 mg/kg of tara extract reduced acrylamide levels by nearly 50%, while 1500 mg/kg resulted in a 97.12% reduction. Sensory evaluation indicated that only crumb color was affected by the addition of the extract (Pedreschi et al., 2018).

Tesby et al. (2018) investigated the effects of powdered anise, thyme, and cumin on acrylamide formation in various bakery products, including Arabic bread, *paton salé*, and *cressina*. Their results indicated that the highest antioxidative activity and greatest AA reduction occurred in paton salé and Arabic bread with anise, and in cressina with cumin. However, the addition of these powders negatively influenced the sensory attributes and overall acceptability across all product types. These findings are consistent with previous studies showing that the antioxidative activity of plant-based additives contributes to inhibiting the acrolein oxidation pathway (Figure 3), thereby reducing AA formation. Additionally, the presence of these antioxidants prevents lipid oxidation and scavenges free radicals, further contributing to AA mitigation (Tesby et al., 2018).

### 6.2 Powders

It is often assumed by researchers and consumers that all plant-based additives exert a reducing effect on AA formation in foods, particularly in bread. However, it is essential to recognize that both the antioxidant capacity and the carbohydrate content of plant tissues play a critical role in determining their overall impact. The addition of certain plant materials can increase the total carbohydrate content of the final product or create favorable conditions for the Maillard reaction, ultimately enhancing AA production.

For instance, chia seeds (*Salvia hispanica L.*) were incorporated into wheat bread formulations at varying concentrations (2%, 5%, 7%, and 10%) to evaluate their effect on AA formation. The findings indicated a non-significant yet higher level of acrylamide in bread samples containing chia seeds compared to control samples. This observation supports the hypothesis that chia seeds contain asparagine, which can be directly converted to acrylamide through the Maillard reaction. Additionally, other amino acids present in chia seeds, such as glutamine, may further promote AA formation. Chia seeds are also rich in lipids, which can enhance acrylamide production through lipid oxidation pathways. Moreover, the presence of divalent ions particularly calcium within chia seed composition may also contribute to elevated AA levels (Galluzzo et al., 2021).

Jaworska et al. (2019) investigated the influence of various vegetable powders on acrylamide formation in cereal crisps. In their study, broccoli, zucchini, and garlic powders were added to one group of cereal crisps, while pumpkin, cinnamon, and ginger powders were added to a second group. The first group demonstrated lower antioxidant potential, which could be related to the relatively high polyphenol content of ginger. However, acrylamide analysis revealed that the addition of pumpkin significantly increased AA levels in the cereal samples. This increase was attributed to the higher sugar content of pumpkin compared with zucchini and broccoli. Although the fructose and glucose contents of these vegetables are relatively similar, pumpkin contains considerably higher levels of sucrose, which may explain its stronger contribution to AA formation (Jaworska et al., 2019).

The impact of plant-based additives on AA formation in bread is strongly influenced by processing parameters, including the type of additive, baking temperature, and baking duration. For example, blueberry, pine nut, rowan, and sea buckthorn powders were incorporated into bakery products at concentrations of 3%, 6%, 5%, and 5%, respectively. Nilova et al. (2019) evaluated the AA content in the resulting products after baking under varying fat contents, temperatures, and times. Their results indicated that shorter baking times and lower temperatures effectively reduced acrylamide levels. Similarly, formulations containing lower amounts of sunflower oil showed reduced AA formation. Among the plant powders tested, sea buckthorn yielded the highest reduction in AA at a baking temperature of 220°C, followed by blueberry, pine nut, and rowan. Interestingly, at a lower temperature of 200°C, blueberry was more effective than sea buckthorn in reducing AA, indicating that the relative efficacy of plant powders may depend on processing conditions (Nilova et al., 2019).

Combining fermentation with plant-based additives has also been shown to effectively inhibit acrylamide formation. Fermentation reduces key AA precursors, particularly free asparagine, through microbial metabolism, and can create synergistic effects whereby additives further neutralize residual precursors or alter conditions to suppress the Maillard reaction. Additionally, fermentation modifies the food matrix, reducing the accessibility of asparagine and sugars, which limits acrylamide formation. Certain additives, such as acids or antioxidants, can enhance these effects by stabilizing reactive compounds or adjusting pH to further inhibit AA production (Zhou et al., 2022).

Bartkiene et al. (2018) investigated the effects of various *Lamiaceae* family plants, including Thymus vulgaris, on acrylamide formation and bread quality. Their study demonstrated that AA formation in wheat bread was significantly influenced by both the type and quantity of plant additives, as well as by the inclusion of scalded flour (SF) or scalded flour fermented with *Lactobacillus plantarum LUHS135* (*SFFLp*). While the addition of T. vulgaris and SF increased AA levels by 2.3-fold compared to bread without SF, combining *SFFLp* with specific plant additives resulted in substantial reductions in acrylamide content. Despite the increase in AA caused by *Thymus vulgaris*, its beneficial effects can still be utilized if paired with fermented flour, which may mitigate its negative impact. The study highlights that optimizing the use of fermented scalded flour in combination with selected plant additives can minimize acrylamide formation while preserving bread quality (Bartkiene et al., 2018).

Overall, plant extracts generally outperform powders in reducing AA formation. The presence of impurities and additional compounds in plant powders can create favorable conditions for the Maillard reaction, inadvertently promoting acrylamide generation. This emphasizes the importance of both additive type and purity when selecting plant-based interventions for AA mitigation.

Although a quantitative meta-analysis was not feasible due to methodological heterogeneity among studies, a critical synthesis was performed. The overall evidence suggests that plant-based additives consistently reduced acrylamide levels in bread, although the magnitude of effect varied by extract type, concentration, and baking conditions. Differences in analytical methods, formulations, and process parameters may explain part of this variability. Future standardized, multi-center studies are needed to confirm these findings and enable meta-analytic integration.

## 7 Comparative analysis

Based on a systematic comparative evaluation of multiple studies, encompassing diverse plant species, incorporation methods, concentrations, and bread products from different regions, our analysis revealed substantial variability in acrylamide modulation. The comparative assessment demonstrates that acrylamide formation is influenced not only by the type and concentration of plant additives but also by processing conditions, bread composition, fermentation method, fat content, and baking temperature. Extracts generally provided more consistent mitigation effects across studies, whereas powders exhibited highly variable outcomes depending on the matrix and processing parameters. These findings underscore that effective acrylamide reduction requires a holistic consideration of additive type, dosage, and the physicochemical and thermal properties of the bread system. Green tea extracts consistently reduced acrylamide formation across multiple studies and products, with reductions ranging from 2.7% to 72.5%. A clear dose-dependent effect was observed, where higher concentrations generally yielded greater reductions. Vine tea and dihydromyricetin extracts in wheat bread exhibited mitigation efficiencies up to 58%, demonstrating their potential as effective natural inhibitors. (-)-Epigallocatechin gallate (EGCG) extracted from green tea also showed significant reductions in white bread, ranging from 31.1% to 38.4%, further confirming the role of catechins in acrylamide mitigation. Spearmint, fennel, and turmeric extracts achieved the highest reduction among all evaluated additives, reaching up to 89% in pita bread, highlighting the efficacy of multi-component plant extracts. Buckwheat seed and sprout extracts displayed moderate reductions (16.7%–27.3%), suggesting a beneficial but less pronounced effect compared to tea-derived compounds. Tara pod extract demonstrated a concentration-dependent mitigation effect, with reductions ranging from 18% to 97% in Chilean bread, illustrating the importance of optimizing dose for maximal effect. Conversely, several plant powders displayed variable or even adverse effects. Thyme powder reduced acrylamide by 98% in Arabic bread but, in fermented wheat bread formulations, resulted in increased acrylamide levels up to 288%. Similarly, Cinnamon, Chia seeds, and garlic powders showed concentration-dependent increases in some cases, with maximum increases reaching 103.5% and 27.7%, respectively. These observations underscore the critical need for careful selection of powder type, concentration, and bread matrix to avoid unintended enhancement of acrylamide formation. Overall, this comparative evaluation highlights that while plant extracts generally provide consistent mitigation effects, plant powders exhibit more complex, matrix-dependent behavior.

## 8 Toxicological relevance

Rosemary, green tea, and vine tea extracts achieved AA reductions ranging from 32% to over 80% by scavenging free radicals, inhibiting lipid oxidation, and interfering with Maillard reaction intermediates (Fu, 2016; Ma et al., 2020; Zhang & Zhang, 2007). From a toxicological perspective, these reductions are relevant because they directly influence the potential dietary intake of AA and its metabolite glycidamide, the latter being implicated in genotoxicity and carcinogenesis. Zhao et al. (2022) investigated the toxicological effects of AA exposure in both healthy and diabetic mice to assess its metabolic and oxidative impacts. The study found that AA administration significantly aggravated glucose and lipid metabolism disorders in diabetic mice by increasing fasting blood glucose and reducing serum lipid levels (TC, TG, LDL-C, HDL-C). Additionally, AA exposure induced oxidative stress and inflammation, evidenced by elevated Malondialdehyde **(**MDA) and Cyclooxygenase-2 (COX-2) levels, decreased antioxidant enzyme activities (SOD, GSH-PX, CAT), and downregulation of Nuclear factor erythroid 2–related factor 2 / Kelch-like ECH-associated protein 1 (Nrf2/Keap1) pathway expression. Overall, the findings indicate that AA exacerbates metabolic dysregulation, oxidative stress, and inflammatory responses in diabetic individuals, suggesting higher susceptibility of diabetics to acrylamide toxicity. Wang et al. (2021) analyzed serum metabolomic changes in rats exposed to AA using UHPLC–Q-Orbitrap HRMS. The study identified significant alterations in metabolites related to the citrate cycle, amino acid, and pyruvate metabolism, indicating systemic metabolic disruption. These changes suggest that AA exposure induces notable biochemical disturbances and may contribute to cardiovascular toxicity. Hou et al. (2021) examined the combined toxicity of T-2 toxin and AA in cell and animal models. Co-exposure produced synergistic cytotoxic effects, significantly increasing liver and kidney damage compared to individual treatments. Mechanistic analysis showed that T-2 toxin suppressed MnSOD expression, while AA reduced catalase, together enhancing oxidative stress. Additives that reduce AA formation may therefore lower the effective dose reaching the gastrointestinal tract and systemic circulation. Moreover, processing conditions, including fermentation, dough pH, and baking temperature, significantly modulate the availability of AA precursors, further impacting exposure levels (Onacik-Gür et al., 2022; Zhou et al., 2022). Notably, some plant powders, such as cinnamon or chia seeds, may paradoxically increase AA formation due to high carbohydrate or aldehyde content, underscoring the importance of selecting appropriate additives to achieve meaningful toxicological benefits. Collectively, these findings establish a direct link between formulation and processing strategies and their potential to reduce acrylamide-associated health risks. By integrating plant-based antioxidants and optimizing processing parameters, it is possible to mitigate AA formation in bread and other baked goods, contributing to lower human exposure and aligning with risk reduction principles outlined by EFSA and WHO.

## 9 Conclusion and future perspective

The results of this systematic review showed that the prevailing approach that the addition of herbal products can only lead to the reduction of AA formation is wrong. An increase in the formation of acrylamide was observed in 60.5% of the samples with powder. Studies showed that the extracts had a better performance in reducing the formation of AA than the powders. But in the powders, an increase in the formation of AA was often observed in compounds such as *Coriandrum sativum, Ocimum basilicum, Origanum vulgare, Carum carvi, Thymus vulgaris, Garlic, Ginger, Chia seeds*. Powders have more impurities than extracts, which can lead to side effects such as increased Maillard reaction. The highest reduction rate was observed for *Tara pod (Caesalpinia spinosa)* extract and the highest effect on the increase of AA formation was observed by *Thymus vulgaris* and cinnamon powder. HPLC method was most used to evaluate the effect of plant-based additives on AA formation. Due to the different effects of herbal additives, a detailed investigation of the amount used and the responses of the changes by meta-analysis studies is required. Optimizing the extract concentration and the changes made for the purification of plant powders can be useful approaches for future studies. Another approach to investigate is the use of a complex of nanoparticles and plant extracts, because each substance can affect different aspects of the mechanism of AA formation. Using native plants of different countries and regions of the world and comparing them with previously used materials can be a suitable approach to obtain effective plant additives. Another approach can be to investigate the effects of plant additives on the conditions of Maillard formation and AA production, such as the effect on pH, water activity and Maillard reaction precursors.

## Declaration of AI-assisted technology in the writing process

The author(s) used ChatGPT for grammar improvement and optimization of the text. The author(s) take(s) full responsibility for the publication content.

## CRediT authorship contribution statement

**Amirhossein Abedini:** Writing – original draft, Methodology, Data curation, Conceptualization. **Mahla Salimi:** Writing – original draft, Methodology, Conceptualization. **Azizollah Zargaraan:** Methodology, Investigation, Conceptualization. **Parisa Sadighara:** Visualization, Methodology, Investigation. **Mahmood Alizadeh Sani:** Writing – review & editing, Investigation, Conceptualization. **Amir M. Mortazavian:** Writing – review & editing, Visualization, Validation, Supervision, Investigation. **Abdorreza Mohammadi:** Writing – review & editing, Visualization, Investigation.

## Declaration of competing interest

The authors declare that they have no known competing financial interests or personal relationships that could have appeared to influence the work reported in this paper.

## Data Availability

The data that has been used is confidential.
